# Current practices of craniospinal irradiation techniques in Turkey: a comprehensive dosimetric analysis

**DOI:** 10.1186/s13014-024-02435-4

**Published:** 2024-04-16

**Authors:** Öznur Şenkesen, Evrim Tezcanlı, Fadime Alkaya, Burçin İspir, Serap Çatlı, Abdullah Yeşil, Ebrar Bezirganoglu, Sezgi Turan, Canan Köksal, Gülay Güray, Emel Hacıislamoğlu, İsmail Faruk Durmuş, Şeyma Çavdar, Telat Aksu, Nurten Çolak, Esra Küçükmorkoç, Mustafa Doğan, Tülay Ercan, Fatih Karaköse, Vildan Alpan, Cemile Ceylan, Gökhan Poyraz, Nilgül Nalbant, Şeyda Kınay, Servet İpek, Namık Kayalılar, Hamza Tatlı, Mingyao Zhu

**Affiliations:** 1 Acıbadem Mehmet Ali Aydınlar University, Istanbul, Turkey; 2 Department of Radiation Oncology, Acıbadem Ataşehir Hospital, Acıbadem Mehmet Ali Aydınlar University, Kayışdağı Cad. No:32, Ataşehir, Istanbul, Turkey; 3 Department of Radiation Oncology, Acıbadem Altunizade Hospital, Istanbul, Turkey; 4 Health Sciences Institute, Istanbul Medipol University, Istanbul, Turkey; 5 Department of Radiation Oncology, Acıbadem Adana Hospital, Adana, Turkey; 6 Radiation Oncology Department, Gazi University, Ankara, Turkey; 7 Department of Radiation Oncology, Medicana Bursa Hospital, Bursa, Turkey; 8 Department of Radiation Oncology, Kolan Hospital, Istanbul, Turkey; 9 Department of Radiation Oncology, Neolife Medical Center, Istanbul, Turkey; 10 Department of Radiation Oncology, Istanbul University Oncology Institute, Istanbul, Turkey; 11 Department of Radiation Oncology, Medikal Park Bahçelievler Hospital, Istanbul, Turkey; 12 Department of Radiation Oncology, Karadeniz Technical University Farabi Hospital, Trabzon, Turkey; 13 Department of Radiation Oncology, Yeni Yuzyıl University Gaziosmanpasa Hospital, Istanbul, Turkey; 14 Department of Radiation Oncology, Medicana Ankara Hospital, Ankara, Turkey; 15 Department of Radiation Oncology, Ondokuz Mayıs University, Samsun, Turkey; 16 Department of Radiation Oncology, Kartal Dr. Lutfi Kirdar City Hospital, Istanbul, Turkey; 17 Department of Radiation Oncology, Anadolu Medical Center, Istanbul, Turkey; 18 Department of Radiation Oncology, Trakya University, Edirne, Turkey; 19 Department of Radiation Oncology, Gayrettepe Florence Nightingale Hospital, Istanbul, Turkey; 20 Department of Radiation Oncology, Koc University Hospital, Istanbul, Turkey; 21 Department of Radiation Oncology, American Hospital, Istanbul, Turkey; 22 Department of Radiation Oncology, Istanbul Onkology Hospital, Istanbul, Turkey; 23 Department of Radiation Oncology, Medipol University Hospital, Istanbul, Turkey; 24 Department of Radiation Oncology, Basaksehir Cam Ve Sakura City Hospital, Istanbul, Turkey; 25 Department of Radiation Oncology, Dokuz Eylul University, İzmir, Turkey; 26 Department of Radiation Oncology, Istanbul University-Cerrahpasa, Istanbul, Turkey; 27 Department of Radiation Oncology, Acıbadem Maslak Hospital, Istanbul, Turkey; 28 Elekta Instrument AB, Barbaros Mah. Begonya Sok. Nidakule, Ataşehir, Istanbul, Turkey; 29 Department of Radiation Oncology, Emory University School of Medicine, Atlanta, GA USA

**Keywords:** Craniospinal irradiation, Secondary cancer risk, Intensity-modulated radiation therapy, Volumetric modulated arc therapy, Tomotherapy, Proton therapy

## Abstract

**Objective:**

This study evaluates various craniospinal irradiation (CSI) techniques used in Turkish centers to understand their advantages, disadvantages and overall effectiveness, with a focus on enhancing dose distribution.

**Methods:**

Anonymized CT scans of adult and pediatric patients, alongside target volumes and organ-at-risk (OAR) structures, were shared with 25 local radiotherapy centers. They were tasked to develop optimal treatment plans delivering 36 Gy in 20 fractions with 95% PTV coverage, while minimizing OAR exposure. The same CT data was sent to a US proton therapy center for comparison. Various planning systems and treatment techniques (3D conformal RT, IMRT, VMAT, tomotherapy) were utilized. Elekta Proknow software was used to analyze parameters, assess dose distributions, mean doses, conformity index (CI), and homogeneity index (HI) for both target volumes and OARs. Comparisons were made against proton therapy.

**Results:**

All techniques consistently achieved excellent PTV coverage (V95 > 98%) for both adult and pediatric patients. Tomotherapy closely approached ideal Dmean doses for all PTVs, while 3D-CRT had higher Dmean for PTV_brain. Tomotherapy excelled in CI and HI for PTVs. IMRT resulted in lower pediatric heart, kidney, parotid, and eye doses, while 3D-CRT achieved the lowest adult lung doses. Tomotherapy approached proton therapy doses for adult kidneys and thyroid, while IMRT excelled for adult heart, kidney, parotid, esophagus, and eyes.

**Conclusion:**

Modern radiotherapy techniques offer improved target coverage and OAR protection. However, 3D techniques are continued to be used for CSI. Notably, proton therapy stands out as the most efficient approach, closely followed by Tomotherapy in terms of achieving superior target coverage and OAR protection.

## Introduction

Craniospinal irradiation (CSI) is a common treatment technique used for medulloblastoma, brain tumors at risk of leptomeningeal spread, and some rare hematological malignancies. The traditional method of delivering CSI involves lateral opposed fields that cover the entire brain and are matched to one or more posterior fields to treat the spine [1]. However, this approach results in dose inhomogeneity, particularly at beam junctions, and exposes non-target tissues anterior to the spinal target volume to a substantial radiation dose. In recent years, technological advancements in radiotherapy have led to the use of modern irradiation techniques such as Intensity modulated radiation therapy (IMRT), volumetric modulated arc therapy (VMAT), and Tomotherapy, which provide better dose sparing and reduce the high radiation dose to healthy tissues.

While these modern techniques increase the volume of the whole body receiving low doses, they also decrease the toxicity of both acute and late side effects by sparing organs at risk (OAR). This reduction in the dose to the intestines and lungs can minimize the frequency and severity of acute side effects such as mucositis, nausea, vomiting, diarrhea, and pneumonia [2].

However, long term toxicity is more of a concern and studies such as the CCSS, CVSS, and RISK register have shown an increased prevalence of cardiovascular diseases, metabolic diseases, and radiation-induced secondary tumors and hypothyroidism as late sequelae of initial irradiation in childhood [3, 4].

To address these challenges, modern irradiation techniques are used to achieve better dose sparing in OARs and more 3D-CRT application of radiation [5, 6]. Several optimization methods have been developed to improve the capabilities of these techniques [7–9]. However, the available evidence regarding the benefits of these techniques remains unclear [10].

In Turkey, both, the conventional 3D-CRT Radiotherapy technique and modern radiotherapy techniques are used for CSI.

The objective of this study was to compare the dosimetric characteristics of various craniospinal radiotherapy techniques currently employed in Turkey in order to assess their advantages, disadvantages, and potential superiority. Our objective was to offer recommendations for minimizing user-based variations among centers that employ the same technique, as well as enhancing the achievable optimal dose distribution using the available resources at radiotherapy centers. To accomplish these objectives, we conducted a comparative analysis of the dose distribution among four craniospinal irradiation (CSI) techniques commonly used throughout the country. Additionally, we examined the variations in dosimetric parameters by comparing them with the proton therapy technique, which is currently unavailable in our country. The primary aim of this study was to enhance the effectiveness and safety of CSI treatment and provide clinicians with valuable recommendations for optimizing treatment plans.

## Materials and methods

For this study, we utilized CT scans from both an adult and a pediatric patient who had received treatment at the Acıbadem Altunizade Radiation Oncology clinic. These scans were employed to identify the target volume and critical organs of the patients. To ensure privacy, the CT scans were anonymized before being distributed in DICOM format to 25 different centers across the country. Additionally, the CT structures were shared with the Emory Proton Therapy center, situated in Atlanta, GA, renowned for its expertise in proton therapy for craniospinal irradiation treatment.

For the purpose of radiotherapy, the adult and pediatric CSI patients were positioned in a supine orientation and immobilized using a thermoplastic head-neck mask and a vacuum bag manufactured by Civco Medical Solutions in Kalona, IA, USA. The CT scans were obtained with a slice thickness of 3 mm.

The contouring of target volumes and OAR was conducted by an experienced radiation oncologist using CT images registered with MRI. The CTV included the whole brain, cranial nerves, and meninges, and was divided into two parts: CTV_brain and CTV_spine. The CTV_spine consisted of the spinal canal from the cerebrospinal fluid to the spinal ganglia, with the lower border being delineated at the caudal extension of the thecal sac.

The planning target volume (PTV) was composed of PTV_brain, PTV_spine, and PTV_total. PTV_brain and PTV_spine were generated with a 5 mm uniform margin around the CTV_brain and CTV_spine, respectively. PTV_total was created as the sum of PTV_brain and PTV_spine. The latest SIOPE guidelines were used to determine the target volume [11].

The organs at risk (OAR) considered in this study included the left and right lenses, left and right eyes, left and right parotid glands, thyroid, larynx, heart, left and right lungs, total lungs, esophagus, left and right kidneys, intestines, and stomach. ART-Plan (Therapanacea) artificial intelligence automatic contouring software was used for automatic segmentation of normal tissues were performed under the control of the physician and dosimetrist. Additionally, the normal tissue volume (NTV) was defined as the external contour of the body excluding the planning target volume (PTV_total). The delineation of the NTV was carried out using the ART-Plan software, and all contours were carefully reviewed and verified by the physician and dosimetrist to ensure precision and consistency.

The study encompassed multiple participating centers, each employing their individual irradiation techniques for treatment planning. The prescription dose for all plans was 36 Gy delivered in 20 fractions of 1.8 Gy. The study requested that at least 95% of PTV_total received 95% of the prescribed dose, while also minimizing dose to critical organs.

The centers utilized a variety of radiation techniques, including 3D-CRT, IMRT, VMAT, and Tomotherapy. The overseas proton center created the plans with the pencil beam scanning technology (Intensity Modulated Proton Therapy or IMPT) method.

### Plan evaluation and statistical analysis

All created plans were uploaded to Elekta ProKnow (Elekta AB) in industry standard DICOM format including DICOM images, RT structure sets, RT plans, and RT doses, and dose volume histogram (DVH) for each plan was recalculated automatically to standardize DVH calculation among different planning systems. For the statistical analysis, scorecards with scoring tables were created based on dose criteria and plans were analyzed accordingly.

The percent volume of PTV_brain, PTV_spine, and PTV_total receiving 95% and 100% of the prescribed dose, as well as the mean and maximum dose values (in Gy) for each target volume, were analyzed for 25 centers and the proton center. The median (min–max) values for each technique were determined. Conformity index (CI) and homogeneity index (HI) values were also calculated for PTV_brain, PTV_spine, and PTV_total using by Elekta Proknow system as below formulas.

$$\begin{aligned} CI & = \frac{{Total\;volume\;\left( {cc} \right)\;covered\;by\;specified\;percent\; \left( \% \right) \;dose\;relative\;to\;specified\;prescription}}{{Volume\; \left( {cc} \right)\;of\;the\;specified\;structure}} \\ HI & = \frac{{Dose\;\left( {Gy} \right) \;covering\;1\% \;of\;specified\;structure - Dose\; \left( {Gy} \right)\;covering\; 99\% \;of\;spesified\;structure }}{{Spesified\;dose\; \left( {Gy} \right)}} \\ \end{aligned}$$

### Treatment planning techniques

Questionnaires containing inquiries regarding the specific technique employed for treatment planning details were distributed to the 25 participating centers, requesting them to provide responses. According to the information reported in the forms, all centers utilizing 3D-CRT techniques implemented divergence matching and moving junction methods. Additionally, four centers indicated the utilization of the Field in Field technique to achieve dose homogeneity.

The centers created brain plans with two lateral fields and one or two spinal plans with a 180° gantry angle. In all IMRT plans, spinal areas were treated with 130°–230° L and R posterior oblique fields added to improve homogeneity and coverage, while keeping OAR doses lower. Centers using VMAT employed avoidance sectors in spinal areas, resulting in arms being left outside of the treatment field and lower normal tissue doses. Users of Tomotherapy technique preferred planning techniques that prevented radiation from arms, using directional block.

## Results

The distribution of techniques employed by the 25 participating centers in the study is as follows: Among them, 7 centers preferred the 3D-CRT technique for pediatric patients, whereas 6 centers utilized it for adult patients. IMRT was the preferred technique for 4 centers in pediatric patients and 3 centers in adult patients. VMAT was employed by 9 centers for pediatric patients and 11 centers for adult patients. Additionally, 5 centers utilizing Tomotherapy employed the helical IMRT technique for both pediatric and adult patients. The planning DVH comparison results for pediatric and adult patients, obtained using the Proknow software, are presented in two distinct groups.

### Pediatric patient

For all techniques employed, including 3D-CRT, IMRT, VMAT, and Tomotherapy, the percentage volumes of PTV_brain, PTV_spine, and PTV_total receiving 95% (34.2 Gy) of the prescribed dose demonstrated V95 values exceeding 98%. The results were consistently similar across the different techniques. Notably, the proton technique achieved precisely 100% V95 for PTV_brain, PTV_spine, and PTV_total, indicating optimal coverage of the target volumes with the prescribed dose. The median (min–max) volume of PTV_brain receiving the full prescribed dose (V100) was highest with the 3D-CRT technique, measuring 96.8 (46.3–99.5). It was followed by the IMRT and VMAT techniques. On the other hand, the lowest V100 value was obtained with tomotherapy, which recorded 86.9 (58.9–95.4). Notably, the 3D-CRT technique exhibited the highest user-based variation in terms of PTV_brain coverage.

For PTV_spinal, the highest median (min–max) V100 value was achieved with the IMRT technique, measuring 95.6 (83.6–98.6). In contrast, tomotherapy, VMAT, and the 3D-CRT technique resulted in lower V100 values, with respective values of 90 (72.9–95.2) for tomotherapy.

Regarding PTV_total, the IMRT technique showed the highest median (min–max) V100 value of 96.6 (69.3–98.7). It was followed by the 3D-CRT and VMAT techniques, while the tomotherapy technique yielded the lowest V100 value of 87.8 (58.0–95.3).

Using the proton technique, V100 values for brain, spine, and total PTV were found to be greater than 97%. The tomotherapy technique exhibited the closest Dmean doses to the ideal values for all PTV target volumes. On the other hand, the 3D-CRT technique yielded the farthest Dmean results for PTV_brain, PTV_spine, and PTV_total. The 3D-CRT technique produced the highest maximum dose values within the PTVs compared to other techniques. In terms of dose distribution conformity (CI results) for pediatric patients, the tomotherapy technique demonstrated the closest adherence to the ideal values for PTV_brain and PTV_spine, while the VMAT technique showed closer conformity for PTV_total.

**Fig. 1:**
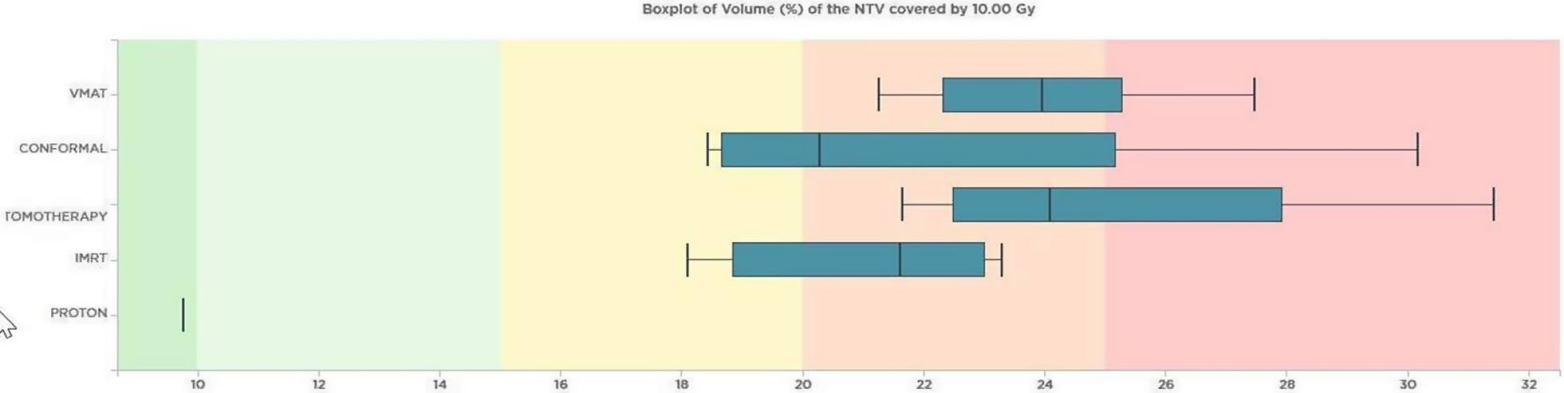
The normal tissue volumes of pediatric patient receiving 10 Gy are shown according to the techniques. It is seen that the lowest normal tissue volume was obtained by 3D-CRT, IMRT, VMAT and Tomotherapy techniques, respectively. It is noteworthy that it is 9.76% with the proton technique

For dose distribution homogeneity (HI index results), the tomotherapy technique outperformed other techniques, providing superior homogeneity for PTV_brain, PTV_spine, and PTV_total. When evaluating the DVH parameters of the 10 Gy volume of healthy tissue outside the PTV in pediatric patients, the 3D-CRT technique exhibited the lowest volume ratio. It was followed by IMRT, VMAT, and Tomotherapy. The rankings for the volumes of normal tissue receiving lower doses (V5Gy and V2Gy) were also consistent with this order. Notably, the results obtained with the proton technique showed significantly lower volumes compared to the other techniques. In Fig. 1, the variation in the volume of healthy tissue receiving 10 Gy across different techniques reveals a notable difference. Specifically, proton therapy exhibited the lowest percentage at 9.76%, differing with other techniques where the lowest was 18.1%, observed in VMAT.

Table 1 presents the results of DVH parameters for PTV V95, V100, Dmean, Dmax, CI, HI, NTV V2, V5, and V10 Gy median (min–max) for pediatric patients.

**Table 1:** Median (min–max) dose values of PTV_brain, PTV_spinal, and PTV_total coverage, Dmean, Dmax, CI, and HI

Pediatric	Conformal (n = 7) median (min–max)	IMRT (n = 4) median (min–max)	VMAT (n = 9) median (min–max)	Tomotherapy (n = 5) median (min–max)	Proton n = 1 value
PTV_brain V95%	**99.8 (98.5–100)**	98.9 (94.3–99.6)	99.2 (97.1–99.9)	99.4 (98.4–100)	100
PTV_brain V100%	**96.8 (46.2–99.5)**	96.8 (66.1–98.8)	94.8 (82.7–96.4)	86.9 (58.9–95.4)	98.8
PTV_brain Dmean (Gy)	37.5 (36.0–41.4)	37.8 (36.1–37.9)	37.8 (36.6–38.1)	**36.5 (36.1–37.3)**	36.7
PTV_brain Dmax	40.4 (38.7–45.7)	40.5 (39.3–41.1)	40.2 (38.9–42.5)	**38.4 (37.9–39.9)**	38.6
PTV_brain CI	1.9 (1.0–2.2)	1.3 (0.9–1.4)	1.22 (1.0–1.3)	**1.2 (0.8–1.3)**	1.4
PTV_brain HI	0.1 (0.1–0.3)	0.2 (0.1–0.2)	0.1 (0.1–0.2)	**0.1 (0.1–0.2)**	0.1
PTV_spine V95%	98.6 (95.5–99.9)	99.3 (97.0–99.8)	**99.4 (97.4–99.9)**	98.7 (97.5–100)	100
PTV_spine V100%	90 (72.9–95.2)	**95.6 (83.6–98.6)**	92.8 (78.6–97.2)	95.2 (54.5–94.7)	97.6
PTV_spine Dmean	38.8 (37.2–39.8)	37.5 (36.7–37.7)	37.5 (36.5–38.2)	**36.9 (36.0–37.4)**	36.5
PTV_spine Dmax	45.0 (41.7–49.0)	41.3 (39.3–42.0)	40.3 (38.7–41.9)	**38.7 (38.6–41.8)**	37.9
PTV_spine CI	8.2 (4.5–9.6)	5.9 (4.0–6.2)	5.4 (4.5–5.6)	**5.2 (3.5–5.5)**	6.0
PTV_spine HI	0.2 (0.2–0.4)	0.2 (0.1–0.5)	0.1 (0.1–0.2)	**0.1 (0.1–0.2)**	0.0
PTV_total V95%	**99.6 (98.2–99.9)**	98.8 (95.2–99.6)	99.3 (97.1–99.7)	99.2 (98.5–100)	100
PTV_total V100%	96.1 (51.1–98)	**96.6 (69.3–98.7)**	95.6 (82. 2–95.7)	87.8 (58.0–95.3)	98.6
PTV_total Dmean	37.8 (36.2–41.1)	37.8 (36.2–37.9)	37.4 (36.6–38.1)	**36.5 (36.1–37.3)**	36.6
PTV_total Dmax	45.0 (41.7–49)	41.3 (39.3–42.0)	40.4 (38.9–42.5)	**38.7 (38.6–41.8)**	38.6
PTV_total CI	1.5 (0.8–1.8)	1.1 (0.7–1.2)	**1 (0.8–1.0)**	1.0 (0.7–1.0)	1.1
PTV_total HI	0.2 (0.2–0.3)	0.8 (0.1–0.2)	0.1 (0.1–0.2)	**0.1 (0.1–0.1)**	0.0
Body-PTV_total V2%	**34.5 (31.1–43.1)**	53.7 (48.9–54.8)	54.3 (39.7–64.9)	60 (55.8–66.1)	13.2
Body-PTV_total V5%	**23.5 (21.6–32.4)**	35.8 (34.8–41.4)	41.7 (32.7–48.4)	45.4 (35.3–49.7)	11.4
Body-PTV_total V10%	**20.3 (18.5–30.2)**	21.6 (18.1–23.3)	24 (21.3–27.5)	24.1 (21.7–31.4)	9.8

Parameters for conformal, IMRT, VMAT, and tomotherapy techniques used in Turkey for pediatric patients, as well as the DVH parameters of proton center. Bold numbers represent the values closest to the ideal

**Fig. 2:**
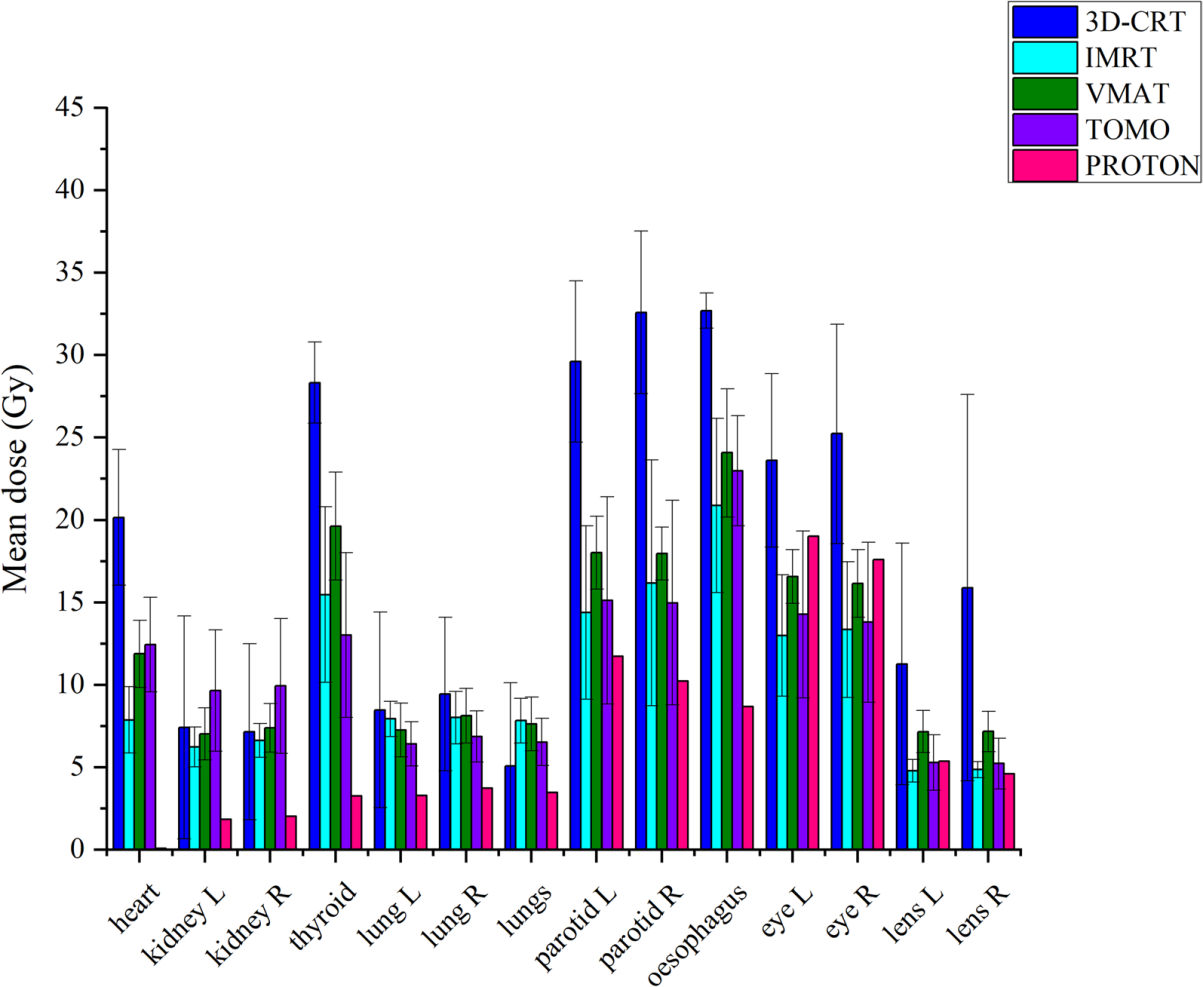
Comparison of OAR mean doses of pediatric patients with 3D-CRT, IMRT, VMAT, tomotherapy techniques. The treatment plans used in our country and proton center. Error bars show the range maximum and minimum

Figure 2 presents a comparison of the Dmean doses of OARs by analyzing the DVH parameters of the techniques used in Turkey and the Emory Proton Center for the pediatric patient. The 3D-CRT technique yielded the highest mean doses for the heart, thyroid gland, left and right parotids, esophagus, and left and right eyes and lenses. On the other hand, the IMRT technique achieved the lowest mean doses, which were closest to the ideal, for the heart, left and right kidneys, left parotid, and left and right eyes and lenses. Tomotherapy resulted in the lowest Dmean doses for the thyroid gland, L and R lungs, and R parotid in total lungs, followed by the VMAT technique. Although the proton technique showed higher doses for the left and right eyes, lenses, and parotids compared to other techniques, these doses remained within acceptable tolerance values. Moreover, the proton technique resulted in significantly lower doses for the heart, left and right kidneys, thyroid, and lungs compared to other techniques.

**Fig. 3:**
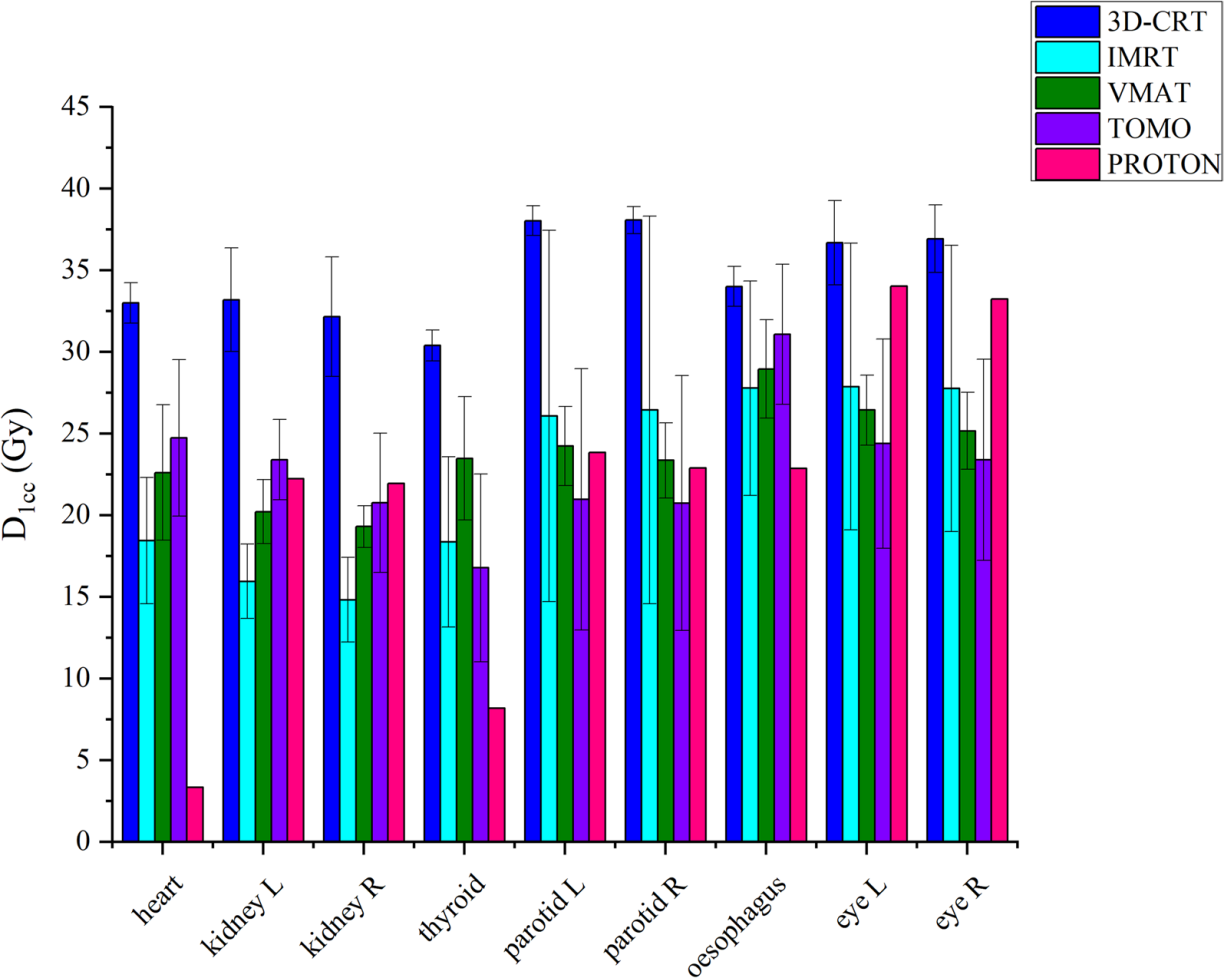
Comparison of OAR 0.1 cc doses of pediatric patients with 3D-CRT, IMRT, VMAT, tomotherapy techniques. The treatment plans used in our country and proton center. Error bars show the range maximum and minimum

Figure 3 shows that D1cc doses in all OARs, the highest 1 cc doses were found in the 3D CRT technique. When the IMRT technique was applied, the lowest doses of 1 cc were obtained in the Kidney L and R, the lowest doses were obtained in the L and R parotids, L and R eyes with Tomotherapy, and the lowest doses were obtained in the heart, thyroid and esophagus with Proton.

**Fig. 4:**
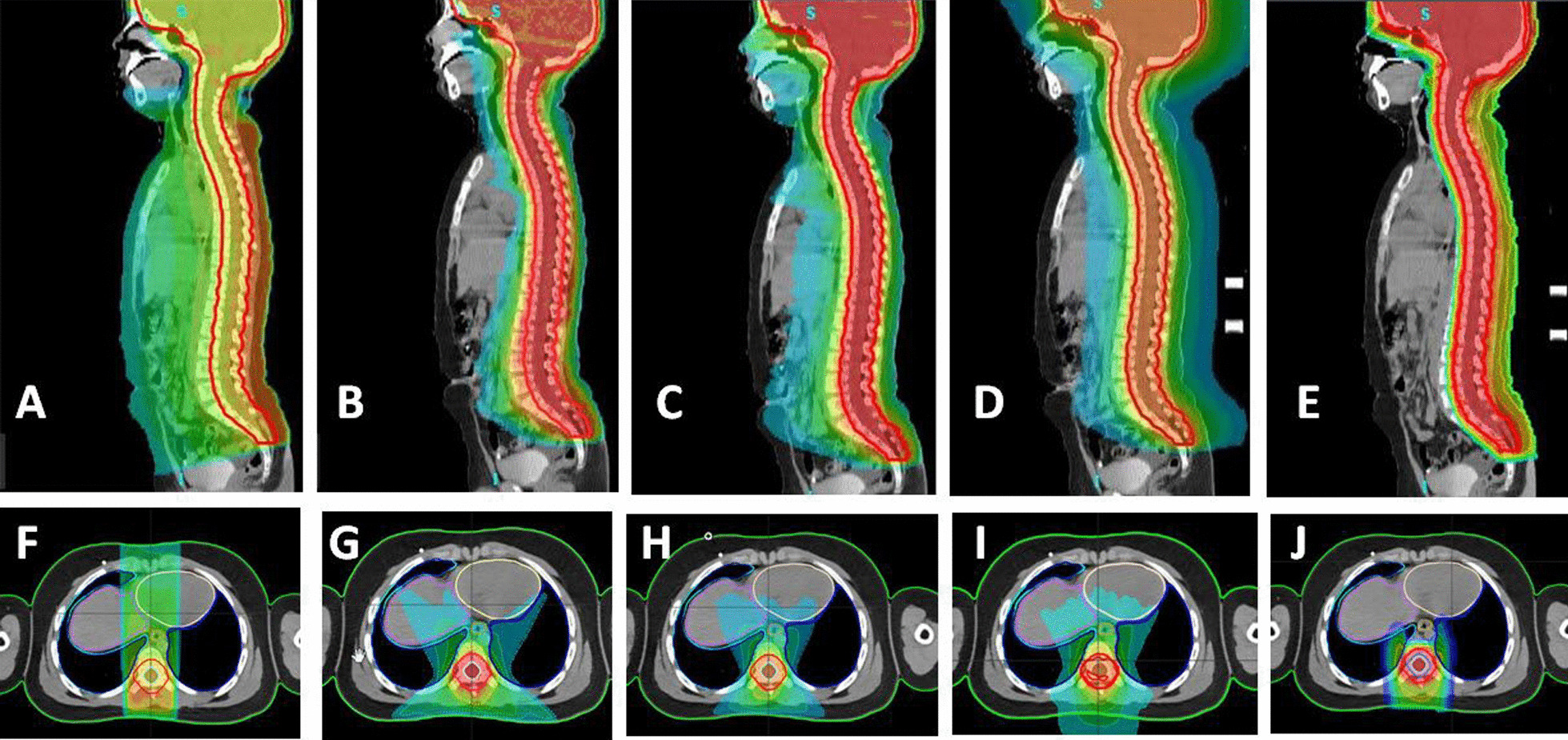
The dose distribution of the 10 Gy volume in the pediatric patient for five techniques. On the body axial and sagital plane was evaluated: sagital plane **A** 3D-CRT, **B** IMRT, **C** VMAT, **D** tomotherapy, **E** proton, axial plane **F** 3D-CRT, **G** IMRT, **H** VMAT, **I** tomotherapy and **J** proton

The dose distribution of the 10 Gy volume in pediatric patients on the axial and sagittal planes for five techniques is illustrated in Fig. 4A–J.

### Adult patient

PTV_brain, PTV_spine, and PTV_total received 95% (34.2 Gy) of the prescription dose, and all techniques achieved V95 > 99%, indicating similar results. Notably, the proton technique achieved 100% V95 for brain, spine, and total PTV.

PTV_brain V100 median (min–max) volume that received the full prescribed dose was obtained by 3D-CRT technique 99.3 (97.3–99.9). This was followed by IMRT and VMAT, while the lowest value was obtained with tomotherapy 92.4 (55.7–95.7). The Tomotherapy technique was found to be the technique with the highest user-based variation in terms of PTV_brain coverage. For PTV_spine, V100 median (min–max) was obtained with the highest Tomotherapy technique 99.6 (97.1–100), respectively, VMAT IMRT and 3D-CRT technique plan result was the lowest 97.1 (87.74–99.7). The 3D-CRT technique was found to be the technique with the highest user-based variation in terms of PTV_spine coverage. for PTV_total V100 median (min–max) results were the highest value by 3D-CRT technique 94.9 (90.6–98.5), followed IMRT and VMAT, and the lowest value was obtained with tomotherapy technique 91.8 (56.5–95.3). With the proton technique, V100 values for brain, spine and total PTV were found to be > 98%. Regarding the Dmean value, the techniques of Tomotherapy, VMAT, IMRT, and 3D-CRT were found to be closest to the ideal value for PTV_brain. Similarly, for PTV_spine and PTV_total, the techniques of Tomotherapy, VMAT, IMRT, and 3D-CRT were ranked in order of being closest to the ideal value. For all PTV volumes, the tomotherapy technique demonstrated Dmean doses that were closest to the ideal values, while the 3D-CRT technique exhibited the furthest results from the ideal for PTV_brain, PTV_spine, and PTV_total. Furthermore, the 3D-CRT technique yielded the highest maximum dose values among all PTVs.

The CI values of PTV volumes were found to be similar in Tomotherapy and VMAT results. In terms of dose distribution homogeneity index (HI), the tomotherapy technique was found to be superior to others, achieving superior results for PTV_brain, spine, and total in adult patients.

**Fig. 5:**
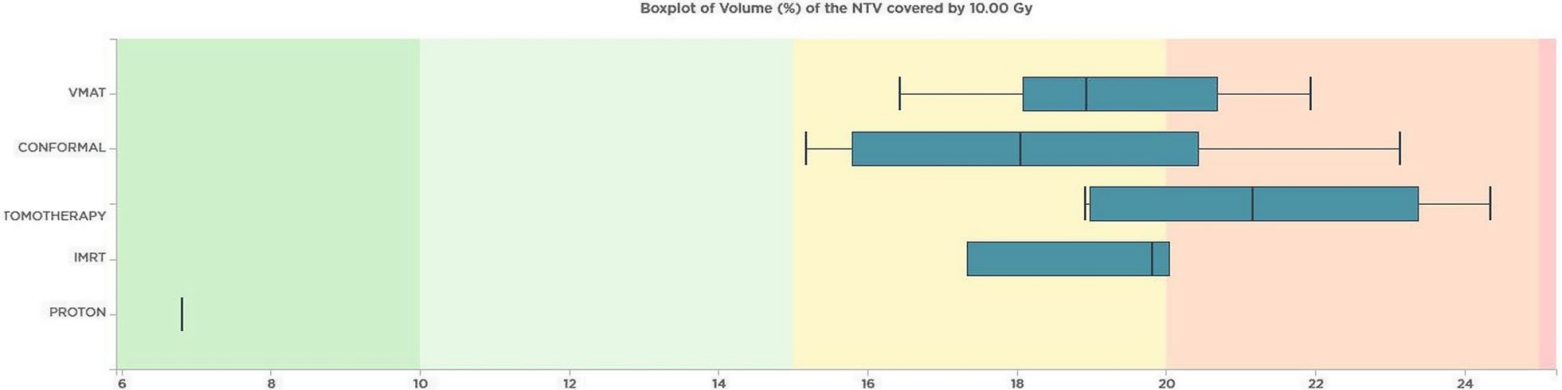
The volumes of normal tissue receiving 10 Gy in adult patient are shown according to techniques. Presented based on the different techniques employed. The results indicate that the 3D-CRT, VMAT, IMRT, and tomotherapy techniques yielded progressively lower volumes of normal tissue. Notably, the proton technique resulted in the smallest volume, measuring 6.81%

The volume ratios of healthy normal tissue receiving a dose of 10 Gy outside the PTV were compared among different techniques. The 3D-CRT technique had the lowest volume ratio, followed by VMAT, IMRT, and tomotherapy techniques, respectively. The lowest volume of healthy normal tissue receiving 10 Gy was found to be 15% for other techniques, while it was only 6.81% for the proton technique. Figure 5 illustrates the variations in volume ratios of normal tissue receiving 10 Gy in the adult patient across different techniques.

The volume ratios of normal tissue volume receiving 2 Gy and 5 Gy were examined. The 3D-CRT technique had the lowest volume ratio, followed by IMRT, VMAT, and tomotherapy techniques, respectively. Table 2 presents the median (min–max) results for adult patients for PTV V95, V100, Dmean, Dmax, CI, HI, NTV V2, V5, and V10. DVH parameters were analyzed for all techniques for adult patients, and the Dmean doses of the OARs were compared.

**Table 2:** Median (min–max) dose values of PTV_brain, PTV_spinal, and PTV_total coverage, Dmean, Dmax, CI, and HI

Adult	Conformal (n = 6) median (min–max)	IMRT (n = 3) median (min–max)	VMAT (n = 11) median (min–max)	Tomotherapy (n = 5) median (min–max)	Proton (n = 1) value
PTVbrain V95%	**100 (99.9–100)**	99 (98–99.7)	99.5 (97.9–100)	99.7 (98.9–100)	100
PTVbrain V100%	**99.3 (97.3–99.9)**	94.4 (70.7–98.8)	92.9 (77.8–99)	92.4 (55.7–95.7)	99
PTVbrain Dmean (Gy)	38.1 (37.2–40.5)	37.9 (36.4–38.1)	37.1 (36.3–37.9)	**36.8 (36.0–37.1)**	36.9
PTVbrain Dmax (Gy)	41.4 (38.9–46.4)	40.6 (39.4–41.5)	40.2 (38.4–43)	**38.6 (37.9–39)**	39.3
PTVbrain CI	2.8 (2.4–3.0)	1.6 (1.1–1.7)	**1.4 (1.1–1.6)**	**1.4 (0.9–1.6)**	1.7
PTVbrain HI	0.1 (0.1–0.2)	0.1 (0.1–0.2)	0.1 (0.1–0.2)	**0.1 (0.1–0.1)**	0.1
PTVspine V95%	97.1 (87.7–99.7)	99.4 (97.8–99.4)	**99.6 (96.4–100)**	**99.6 (97.1–100)**	100
PTVspine V100%	84.9 (69.5–96)	**95.4 (67.7–97.0)**	93.0 (72.6–98.5)	87.5 (58.5–94.6)	99.1
PTVspine Dmean (Gy)	38.7 (37.6–40)	37.8 (36.3–38)	37.4 ( 36.3–38.1)	**36.7 (36.1–37.2)**	36.8
PTVspine Dmax (Gy)	46.9 (44–50.6)	41.5 (39.0–42.1)	40.5 (39–45.1)	**39.1 (37.8–39.8)**	38.5
PTVspine CI	6.3 (5.6–7.0)	3.6 (2.5–3.8)	**3.3 (2.5–3.6)**	**3.3 (2.1–3.6)**	3.9
PTVspine HI	0.3 (0.2–0.4)	0.2 (0.1–0.3)	0.1 (0.1–0.2)	**0.1 (0.1–0.2)**	0.1
PTVtotal V95%	99.4 (97.8–100)	99.1 (98.4–99.2)	99.4 (97.8–100)	**99.6 (99–100)**	100
PTVtotal V100%	**94.9 (90.6–98.5)**	94.7 (69.8–98.3)	93.9 (76.2–98.5)	91.8 (56.5–95.3)	99.0
PTVtotal Dmean (Gy)	38.3 (37.7–40.0)	37.9 (36.3–38.0)	37.0 (36.3–38)	**36.7 (36.0–37.1)**	36.9
PTVtotal Dmax (Gy)	46.9 (44–51)	41.5 (39.4–42.1)	40.6 (39–45.1)	**39.1 (37.9–39.8)**	39.3
PTVtotal CI	1.9 (1.7–2.1)	1.1 (0.8–1.2)	**1.0 (0.8–1.1)**	**1 (0.6–1.1)**	1.2
PTVtotal HI	0.3 (0.2–0.3)	0.2 (0.2–0.1)	0.1 (0.1–0.2)	**0.1 (0.1–0.1)**	0.1
Body-PTVtotal V2%	**30.4 (27.1–37)**	48.7 (46.4- 50.2)	52.7 (37.2–71.7)	61.1 (57.8–70.8)	9.4
Body-PTVtotal V5%	**20.0 (17.8–25.9)**	34.2 (31.9–35.4)	36.8 (28–48.3)	43.9 (37.8–49.4)	8
Body-PTVtotal V10%	**10.1 (15.2–23.1)**	21.2 (18.9–24.4)	18.9 (16.4–21.9)	19.8 (17.3–20.1)	6.8

Parameters for conformal, IMRT, VMAT, and tomotherapy techniques used in Turkey for adult patients, as well as the DVH parameters of proton center. Bold numbers represent the values closest to the ideal

The highest Dmean doses were observed in the plans using the 3D-CRT technique for the heart, thyroid gland, L and R parotid gland, esophagus, L and R eyes, and R lens. On the other hand, the lowest Dmean doses for L and R lung, total lung, right and left kidneys were obtained with the 3D-CRT technique. The tomotherapy technique yielded the lowest OAR Dmean doses, which were closest to the proton technique results, for the L kidney and thyroid gland. In terms of specific organs, the IMRT technique resulted in the lowest doses for the heart, R kidney, L and R parotid gland, esophagus, L and R eyes, and lenses.

**Fig. 6:**
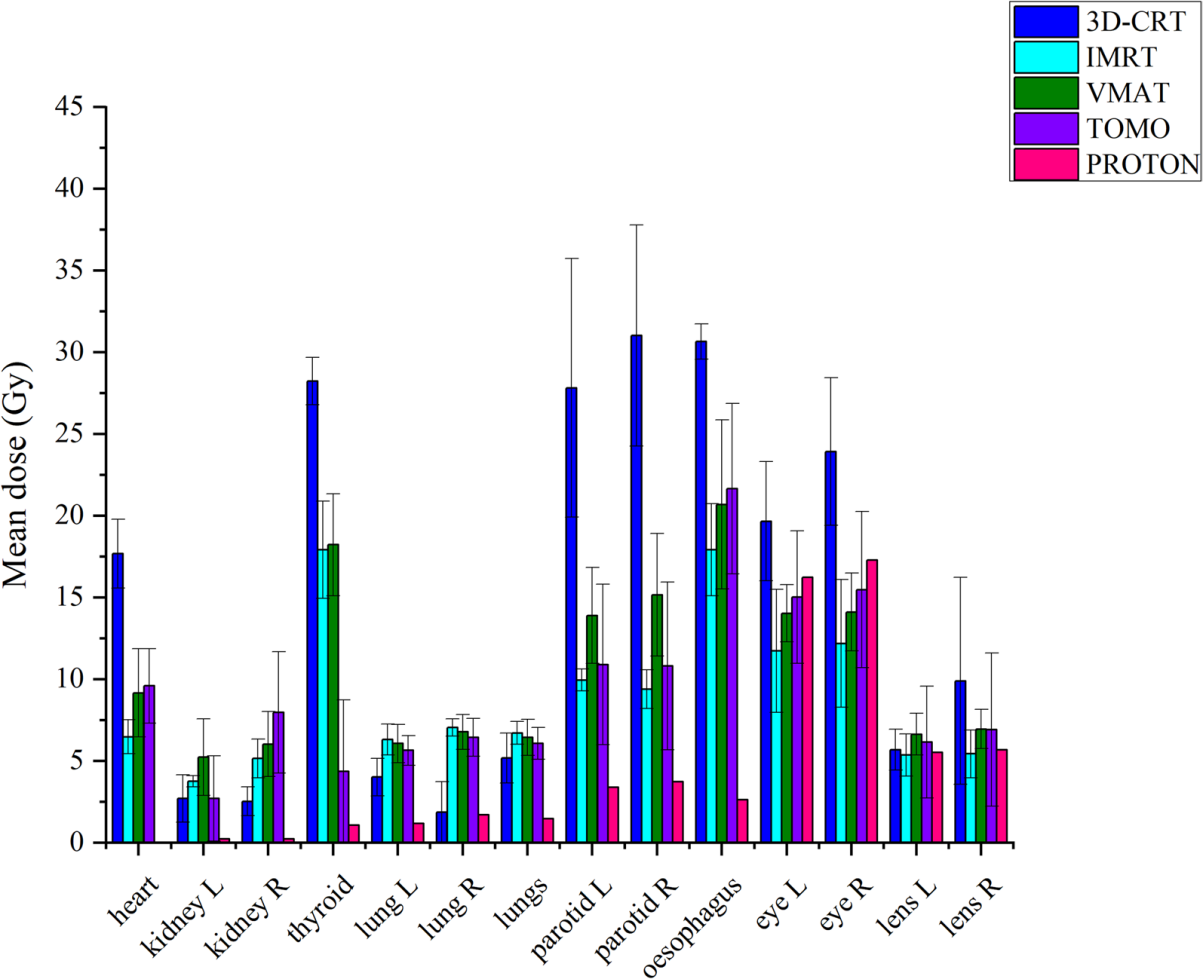
Comparison of OAR mean doses of adult patients with 3D-CRT, IMRT, VMAT, tomotherapy techniques. The treatment plans used in our country and proton center. Error bars show the range maximum and minimum

The results comparing the Dmean doses of OARs by examining the DVH parameters for each technique in adult patients are presented in Fig. 6.

The analysis of the proton technique revealed that while the doses to the L and R eyes appeared higher compared to the IMRT, VMAT, and Tomotherapy techniques, they remained within acceptable tolerance levels. Conversely, notable reductions in dose were observed for the heart, L and R kidneys, thyroid, L and R parotids, esophagus, and lung when utilizing the proton technique.

**Fig. 7:**
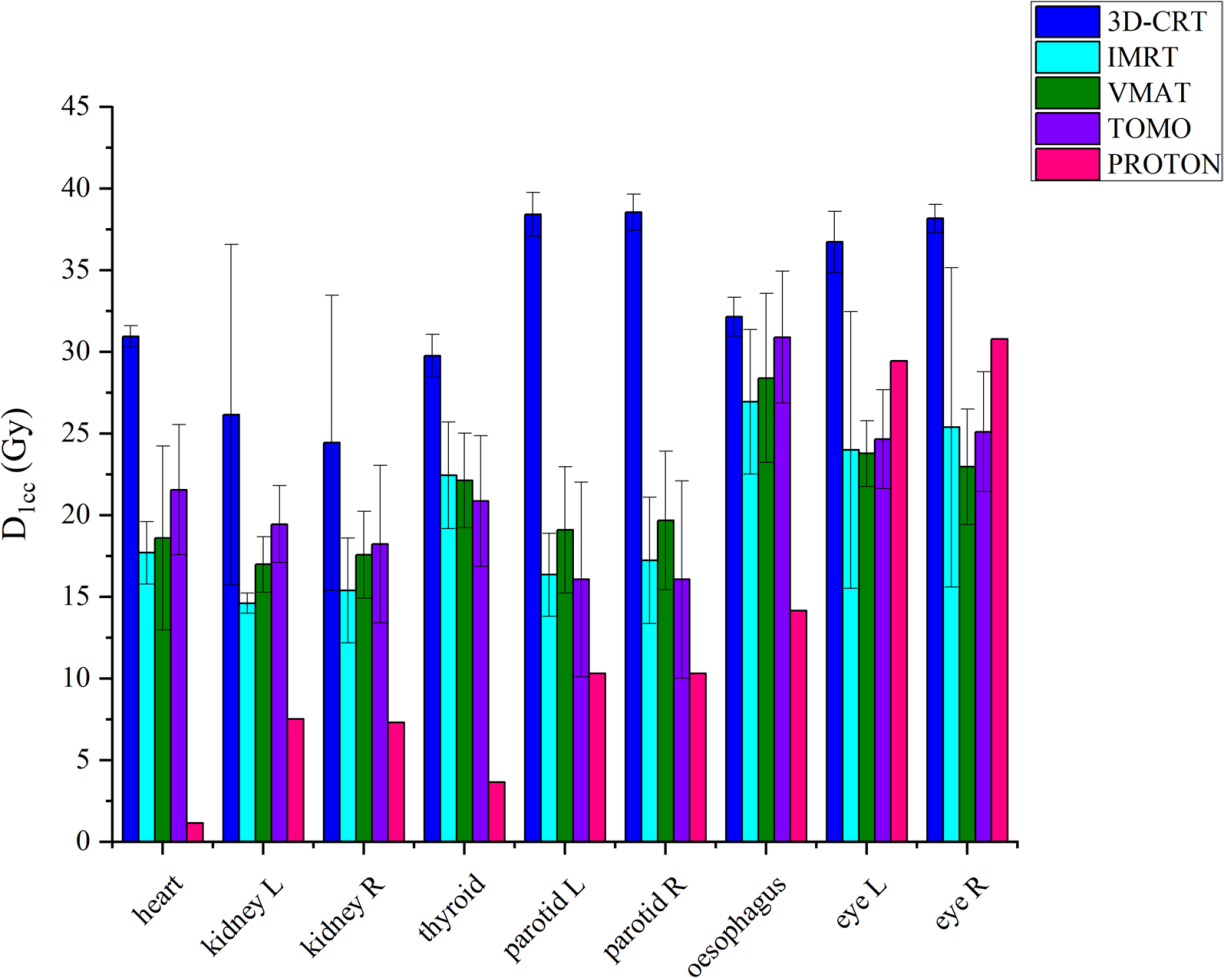
Comparison of OAR 0.1 cc doses of adult patients with 3D-CRT, IMRT, VMAT, tomotherapy techniques. The treatment plans used in our country and proton center. Error bars show the range maximum and minimum

Figure 7 illustrates that the D1cc doses in all OARs for adult patient indicate the highest 1 cc doses in the 3D CRT technique. In contrast, the lowest 1 cc doses for the eyes (both left and right), heart, left and right kidneys, thyroid, as well as the left and right parotid glands and esophagus, were achieved with the proton technique.

In the plans generated using the 3D-CRT technique, it has been noted that the maximum dose values exceed the acceptable limits, ranging from 115 to 125% as a result of normalization performed to increase the coverage.

## Discussion

In our study conducted in Turkey, we discovered that the 3D-CRT technique remains widely utilized, despite the availability of advanced LINACS and treatment planning systems capable of implementing IMRT and VMAT techniques. Clinics with advanced technology still preferred the 3D-CRT technique due to its minimal low-dose bath volume. However, this preference for the 3D-CRT technique resulted in less homogeneous and lower coverage in the target volume, especially in the spinal region. Further examination of the best dose distribution plans using the 3D-CRT technique revealed the utilization of divergence-compatible angles and the moving junction technique. Additionally, incorporating Field in Field helped achieve a more homogeneous dose distribution in high-dose regions. In comparison to the 3D-CRT technique, advanced techniques such as IMRT, VMAT, and Tomotherapy demonstrated superiority in reducing doses to critical organs, except for the lungs and kidneys in terms of OAR doses. There have been several studies comparing craniospinal irradiation technics [7, 12, 13]. Sun et al.'s comparison of Helical Tomotherapy, IMRT, and VMAT in the treatment of CSI, have found that HT offers superior outcomes in terms of PTV conformity, PTV homogeneity, and critical OAR sparing [14].

We also conducted a comparison of proton treatment plans for both adult and pediatric patients at the proton center included in our research with the treatment plans of the four techniques commonly employed in our country. The results highlighted the superiority of proton treatment in maintaining target volume coverage and reducing doses to OAR as well as low dose area. The findings of our study unequivocally highlight the significant value associated with establishing a proton center in our country, particularly for pediatric patients. The importance of offering these opportunities to our patients in the forthcoming years becomes evident.

Separate analyses for adult and pediatric patients were conducted due to the heightened risk of secondary cancer and potential side effects in pediatric cases, underscoring the need to minimize normal tissue dose.

We conducted a comparative analysis of techniques between adult and pediatric patients to determine the approach yielding the lowest healthy tissue dose and to investigate whether center preferences vary based on the patient's age. Among the 11 centers employing the VMAT technique for adults, one used IMRT for pediatric patients, and another used the 3D-CRT.

The distinction in planning requirements between age groups prompted the use of different methods, highlighting the impact of age-specific factors on optimal treatment strategies. Due to the length of the spinal area, pediatric patients can be treated with a single spinal area, whereas in adult patients, two adjacent areas are needed, necessitating various methods employed by centers (such as moving junction, etc.) to compensate for dose distribution at the area junction.

In a study conducted by Seravalli et al. comparing different CSI techniques applied in various European countries, modern photon techniques were found to have superior spinal PTV conformity and homogeneity indices, with lower doses for thyroid, heart, esophagus, and pancreas than 3D-CRT technique. When proton technique was used, there was a more than 10 Gy dose reduction in parotid glands, thyroid, and pancreas compared to photon techniques. However, the study also reported wide dosimetric differences between centers using the same technique in proton applications [9]. Our study found that the 3D-CRT technique, although widely used in Turkey, has limitations in achieving optimal target coverage and dose homogeneity, particularly in the spinal region; this finding is consistent with previous studies that have reported the same limitations [14]. We observed that advanced techniques such as IMRT, VMAT, Tomotherapy, and Proton Therapy offer superior target coverage and dose homogeneity compared to the 3D-CRT technique. This finding is in line with the study by Seravalli et al. which reported that modern photon techniques offer superior spinal PTV conformity and homogeneity indices compared to the 3D-CRT technique, and proton therapy provides a significant dose reduction in critical organs compared to photon techniques [9]. While the dosimetric outcomes of this study favor proton therapy and tomotherapy, it's noteworthy that 3D-CRT techniques were the most preferred. However, it's important to highlight that there was a notable variation in maximum doses observed among centers employing similar 3D-CRT techniques.

Pollul et al. conducted a study comparing different VMAT techniques, namely partial arc, full arc, and avoidance sector, with 3D-CRT plans. The objective was to assess their effectiveness in reducing dose exposure to OARs such as the heart, thyroid, or gonads to minimize the likelihood of late complications. The results demonstrated that the Short Partial Arc VMAT_AVD technique successfully reduced dose exposure to radiosensitive OARs when compared to the 3D-CRT method, resulting in a lower probability of late complications [10]. In our own study, all centers employing the VMAT technique utilized the avoidance sector. While the VMAT technique achieved comparable target volume coverage in dose distributions, particularly in pediatric patients, it notably outperformed the 3D-CRT technique in reducing doses to OARs.

Furthermore, our study revealed that proton therapy provides excellent target coverage and low OAR doses compared to all four techniques used in our country. This finding is consistent with previous studies that have reported the superior dosimetric properties of proton therapy in reducing the dose to critical organs [15].

The risk of second primary cancers, particularly associated with high or very high radiation doses, is attributed to the repopulation of heavily irradiated tissues by surviving stem cells, some of which may have undergone malignant transformation due to radiation exposure [16]. While the exact mechanism remains elusive, various models have been proposed. Understanding these mechanisms is critical, especially for evaluating the risks tied to advanced radiation treatment methods like IMRT and VMAT. These techniques deliver high doses to the target volume while exposing relatively large volumes of healthy tissue to low to moderate doses [17]. Additionally, treatments employing protons or heavy ions instead of photons offer precise targeting of treatment volumes, thereby reducing healthy tissue exposure [18]. However, they may introduce other concerns, such as the production of secondary neutrons during treatment [18, 19].

Assessing how the risk of second primary cancers changes with radiation dose is a complex undertaking, primarily due to the diverse treatment protocols used over the years, including variations in fractionation and concurrent chemotherapy [16]. Notably, Dörr and Herrmann [20] discovered that nearly 60% of second cancers developed within tissues corresponding to the 'penumbra' of the initial radiotherapy volume, receiving a dose less than 6 Gy, while about 35% developed at doses between 10 and 30 Gy. Additionally, research has explored the interaction between cancer treatment and genetic mutations in DNA repair genes, providing insights into the risks associated with specific radiation doses and genetic profiles [21]. Their findings indicated that mutations in homologous recombination genes were significantly associated with an increased rate of second primary female breast cancer, particularly among survivors who had received chest doses greater than or equal to 20 Gy. Mutations in nucleotide excision repair genes were associated with second primary thyroid cancer among survivors who had received neck doses greater than or equal to 30 Gy [21]. The risk of second primary cancers following cumulative high radiation doses varies depending on the exposed tissue. Aside from a reduction in the risk of thyroid gland cancer, the risk of second primary solid cancers generally appears to increase linearly with an escalating cumulative tissue dose, particularly within a dose range of tens of gray [20].

Proton therapy has shown promising results in reducing the risk of secondary malignancy compared to photon techniques. This is because protons have a characteristic depth-dose distribution that allows for more precise targeting of the tumor volume while sparing the surrounding healthy tissues [22]. However, the use of proton therapy also requires careful consideration of the risks associated with secondary neutrons, which can contribute to the risk of secondary malignancy [23]. While Mirabell et al. proposed that proton beam therapy might play a role in substantially reducing secondary cancers among children and adolescents undergoing treatment, it's important to note that Upadhyay et al. suggested that the risks associated with secondary cancers could be comparable with both techniques [19, 22].

There is limited information available regarding the use of specific techniques in craniospinal irradiation (CSI) in low-income countries. However, Abdel-Wahab et al. analyzed the availability and accessibility of radiotherapy services in low- and middle-income countries (LMICs), where resources and infrastructure may be limited. The study provided a comprehensive analysis of the radiotherapy landscape in LMICs, the use of more advanced techniques such as IMRT or VMAT was limited due to the lack of necessary equipment and human resources [24]. Although our country falls into the middle-income category, over the last decade it has made significant progress in terms of radiation therapy equipment, with several centers equipped with state-of-the-art technology. We believe concerns over secondary cancers prompted a preference for 3D-CRT treatment techniques in clinics, however considering current research, we recommend centers equipped with modern technologies like IMRT and VMAT to adopt these techniques. The Dutch 3D study, an ongoing endeavor, encompasses 126 patients who have undergone craniospinal RT for CNS tumors, with a predominant focus on medulloblastoma (90 patients), as well as other histologies and treatment sites. This study aims to compile and standardize digital radiotherapy records from various multi-center resources, with the ultimate objective of enabling the future calculation of organ-specific radiation doses for childhood cancer survivors who received treatment during the 3D era [25]. Therefore, while 3D-CRT technique may have advantages in terms of reducing the volume of low dose bath, the use of advanced techniques such as IMRT, VMAT, and proton therapy can also reduce the risk of secondary malignancy by minimizing the dose to the surrounding normal tissues [22]. However, the risks associated with secondary neutrons in proton therapy should also be carefully considered when selecting the appropriate treatment modality for craniospinal radiotherapy. Clinicians should weigh the potential benefits and risks of each treatment option on a case-by-case basis, taking into account the patient's medical history, age, and other individual factors. Despite limitations, our study provides valuable insights into the current practice of craniospinal radiotherapy in Turkey and highlights the need for centralization of RT services to more experienced centers using advanced techniques.

## Conclusion

In conclusion, our study suggests that advanced techniques such as IMRT, VMAT, and proton therapy offer superior target coverage and dose homogeneity compared to the 3D-CRT technique. Therefore, we recommend leveraging advanced technology to overcome the challenges in organizing field overlap areas and reducing OAR doses when devices that apply IMRT and VMAT techniques are available. Furthermore, establishing a proton center in our country is highly valuable, especially for pediatric patients, and provides excellent opportunities for reducing the dose to critical organs. Centralization of radiation therapy services to centers using advanced techniques with more expertise should be considered especially for pediatric patients undergoing CSI.
